# Monitoring Technology of Abnormal Displacement of BeiDou Power Line Based on Artificial Neural Network

**DOI:** 10.1155/2022/7623215

**Published:** 2022-08-31

**Authors:** Jingbo Yang, Yihan Chen, Jiarong Yu, Zheng Zhou, Yanna Guo, Xingye Liu

**Affiliations:** Wuxi Guangying Group Co., Ltd., Wuxi 214000, Jiangsu, China

## Abstract

In the practice of power line engineering, navigation and positioning technology is often used in the fields of information collection and analysis, optimized line design, and deformation monitoring. Compared with traditional measurement technology, it has the characteristics of high precision and high reliability. In order to realize the measurement of abnormal displacement of power lines, improve the efficiency and quality of monitoring, and reduce the occurrence of faults, firstly, this study introduces the basic theory of artificial neural network (ANN). The core algorithm of the ANN-BP (back propagation) neural network has been improved. The improved algorithm is used to improve the BeiDou Navigation Satellite System (BDS). The improved and the unimproved BDS are used to solve the collected related data. The results show that the geometric dilution of precision (GDOP) values obtained by conventional BDS are small, all within the range of less than 4. After the introduction of the BP neural network into the system, the geometric space distribution of positioning satellites is improved, the GDOP is reduced, the reliability and availability of satellite positioning are enhanced, and the accuracy requirements are met. The accuracy of the measured data positioning results of the two systems has reached the cm level. There is not much difference between the processing results of the two modes. Among them, the *Z* direction accuracy has the largest difference, which is 2.5 cm. The introduction of the BP neural network has improved the spatial combination structure, and the positioning results in the three directions of *X*, *Y*, and *Z* are all better. From the perspective of root mean square (RMS), the RMS fluctuation of the simulation results obtained by observing the conventional BDS is large. The RMS value of BDS displacement based on the BP neural network is smaller, and the change is gentle. With the increase in the number of epochs and the increase in the number of simulations, its value is also more convergent. These data show that the quality of BDS observations based on the BP neural network is significantly better. These contents will effectively improve the monitoring accuracy and operational reliability of the system and have important practical significance and application value.

## 1. Introduction

The power line is one of the main facilities of the power system. Its normal and stable operation is the basis for ensuring the safety and trouble-free of the power system. Power towers and power transmission and transformation lines distributed in the wild may experience abnormal displacement and other phenomena due to the interference of external factors, resulting in serious accidents. Such accidents will have very adverse effects on the social economy and people's lives. The related research on abnormal displacement monitoring method of power lines has attracted extensive attention from scholars [1]. The purpose is to analyze the principle of the BeiDou dual-mode positioning system in the application of abnormal displacement monitoring of power lines, study the conversion technology of different reference systems, and unify the two-time systems and coordinate systems. The main sources of error are analyzed. Electromagnetic wave delay models for the ionosphere and troposphere are established. The satellite and receiver clock error calculation model eliminates errors through mathematical models such as double difference and improves the accuracy of observation data.

At present, there are two main early warning methods for transmission line status: the first one is the early warning method based on big data, deep learning, and other computer technologies. Hubei Electric Power Co., Ltd. adopted the icing prediction model based on extreme learning machine and the icing prediction model based on support vector machine (SVM) to predict the icing of transmission lines. The experimental comparison results show that the prediction model based on SVM is better [2, 3]. The second is to divide the status level according to the evaluation result of the transmission line and take corresponding measures to carry out early warning according to the status level. The improved fuzzy comprehensive evaluation (FCE) is used for early warning of conductor galloping in transmission lines. Based on the optimal theory to optimize the weight, a comprehensive evaluation model is constructed in combination with the principle of fuzzy mathematics to improve the influence of the subjective weight of experts in the traditional fuzzy hierarchical evaluation. The rapid development of smart grids brings higher maintenance costs and greater scalability of transmission lines. An effective and safe power line monitoring system has become a bottleneck restricting the intelligentization of the power grid. In response to this problem, Fan et al. [4] proposed a new method for intelligent monitoring of power grids based on inspection robots, wireless sensor networks, and fault-tolerant sensor networks to achieve low cost, energy-saving, elastic, and remote monitoring. Through robotic fault-tolerant sensor networks, smart grids can detect faults in transmission lines and assess the operating state of the grid. Natural disasters and physical failures of overhead transmission lines have a severe impact on the grid, such as mechanical failures, power losses, line capacity reductions, and voltage drops. Jeyanthi et al. [5] pointed out that these adverse effects can be reduced by implementing an appropriate monitoring system. These methods have achieved certain results in the application process, but there are still some shortcomings and deficiencies. How to improve the accuracy of abnormal displacement monitoring of BeiDou power lines is still an issue that has been explored and discussed in academic circles.

In the practice of power line engineering, navigation and positioning technology is often used in the fields of information collection and analysis, optimized line design, and deformation monitoring. Artificial neural network (ANN) is introduced into BDS. Firstly, the basic theory of ANN is introduced. The core algorithm of ANN, the back propagation (BP) neural network, is improved. An improved algorithm is used to improve the BDS. The improved and unimproved BDS are used to solve the collected correlation data. The innovation lies in the application of ANN to the abnormal displacement monitoring technology of power BeiDou lines. The improved and unimproved BDS are used to solve the collected related data and achieve better monitoring accuracy. These conclusions provide a reference for the monitoring of abnormal displacement of power lines to realize the measurement of abnormal displacement of power lines, improve the efficiency and quality of monitoring, and reduce the occurrence of faults.

## 2. Research-Related Theories and Experimental Design

### 2.1. Line Abnormal Monitoring

**Figure 1:**
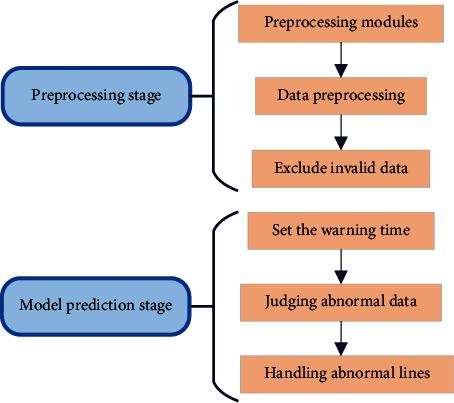
Two stages of line data identification.

Identification of abnormal data: the identification range of abnormal data of line status includes data information related to the line connected to the big data analysis system. The identified data are identified as the corresponding data state estimate. Line data identification should be carried out in two stages, namely, the data preprocessing stage and the model prediction stage. The data preprocessing stage is processed with the help of rules to eliminate abnormal situations caused by problems such as line connection, data association, and line correspondence. Such cases are exceptions caused by artificial reasons, and the abnormal state is constant before modification. The model prediction stage mainly uses machine learning algorithms to predict whether the line will be abnormal. The object of its prediction is the data itself, which has nothing to do with the overall grid [6–8]. The specific identification of the two stages is shown in Figure 1.

In Figure 1, in the preprocessing stage, the set preprocessing module is used to process the system's data to realize the function of eliminating invalid data. The content of abnormal data identification includes univariate data identification and cross-data identification. Univariate data identification is processed, including incomplete data, invalid data, and abnormal data quality. Cross-data identification needs to consider the overall network of the circuit, including the inability of line points to correspond to lines, the judgment of whether the connection point is floating, the position of the circuit breaker or knife switch does not correspond, and the equipment connection point is incomplete. The relationship is missing, or the filling is incorrect, etc. The model prediction stage, based on the machine learning model, predicts the state of the line for a period, including abnormal or normal. Model prediction must have a time advance, and a reasonable early warning time needs to be set to facilitate the staff's response, processing, and repair work. In the process of data mining, judging whether the data are abnormal can be processed from the data distribution. For example, the clustering algorithm can be used to judge the samples far from the cluster center as abnormal data. In a project, anomaly states are different from purely data-based anomalies in data mining. The abnormal early warning of the project is to facilitate the work of monitoring and maintenance personnel, to deal with abnormal lines in a timely manner, and to prevent the waste of manpower and physics caused by abnormal conditions. Electricity anomaly is used as the criterion for abnormality judgment. The abnormal situation identification can be processed by using the three-phase voltage value and the three-phase current value of electric power, and different processing can be performed according to the voltage level of the line. According to China's national network power data standard specification, there are some criteria for judging whether the power equipment is abnormal: for power system equipment of 35 kV and above, the absolute value of the positive and negative deviation between the measured data value and the rated value shall not exceed 10%. The deviation between the measured value and the rated value of the power system equipment data of 10 kV and below shall not exceed 7%. Since the voltage levels of different lines and the same line at different times will be changed and adjusted, the data of different voltage levels should be processed separately during the processing.

**Figure 2:**
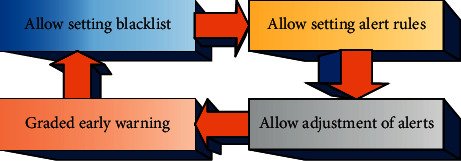
Setting of early warning preliminary conditions.

Real-time early warning of abnormal state: a high-performance monitoring system should have the function of early warning in minutes. The alarm frequency of the system is consistent with the data collection frequency, which is performed once every minute. The prediction period is set to 30 minutes, that is, to predict whether the line will be abnormal after 30 minutes. The reason for being set to 30 minutes comprehensively considers the balance between the prediction accuracy and the response time and processing time of the staff after the warning [9]. If the prediction time is too short, although the accuracy rate can be improved, the processing time for the staff is short, and the staff may not be able to take timely measures to troubleshoot the fault. On the other hand, if the setting time is too long, although there is enough time for processing, the prediction accuracy is low, and the false-positive rate and coverage rate will be affected. In addition, it should be possible to set early warning rules, and for the prediction set with an abnormal prediction probability greater than the threshold, only the set abnormal situation is displayed. Based on generalized early warning, the customization of early warning objects is allowed; that is, monitoring personnel are allowed to set early warning conditions [10]. The preliminary conditions are set as shown in Figure 2.

In Figure 2, the first step in setting the preliminary warning conditions is to allow the blacklist to be set. The actual use object of the prediction system is the monitoring staff of various places. They have a better understanding of the warning line and are more aware of the line maintenance and stoppage time. So, they are allowed to set a blacklist. After the prediction results are filtered out of the lines in the blacklist, other abnormal lines are aggregated and displayed to the user. The second step is to allow the setting of alert rules. Unlike a blacklist that is specific to each line, an alert rule is a collection of several alert conditions. If the warning condition is more than four times within five minutes, the warning level is set to A-level warning. If the number of warnings is greater than two and less than 4, it is set as a B-level warning. The first warning is set as a C-level warning. Such warning rules can classify warnings and set higher levels for more serious warnings, which is convenient for users to deal with in a timely manner. The third step is to allow adjustment of the early warning value. The prediction result of the prediction model is the corresponding abnormal probability. For the line whose abnormal probability is greater than a certain threshold, an early warning is performed, and the initial value of the early warning value can be set to the optimal value. Additionally, dynamic adjustment of the threshold value is allowed. There is a trade-off between forecast accuracy and forecast coverage. The fourth step is graded early warning. Like the second step, early warning is carried out according to the conditions and levels of the predicted probability. Like the second step, the early warning is divided into conditions and levels according to the predicted probability. The difference is that the second step allows user-defined rules to be limited. The graded early warning refers to the predicted abnormal probability value of the line, and the line with a higher abnormal probability value should be dealt with first.

**Figure 3:**
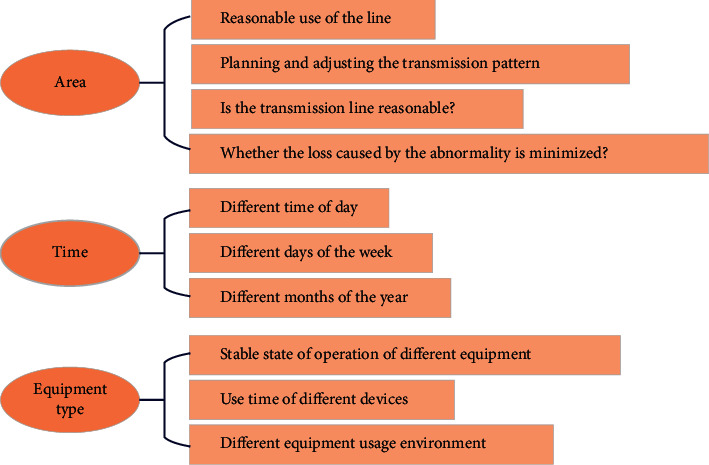
Subtotal summary of forecast data.

Statistical analysis of historical abnormal data: the abnormal prediction of line status is classified and archived according to region and time, which is convenient for data analysts to conduct statistical analysis. The statistical analysis of historical data is mainly to compare and analyze the predicted state of the transmission line in the statistical period between the measured state value and the corresponding state predicted value [11]. The forecast situation and the monitoring situation are analyzed. Statistical analysis results will be displayed in the form of lists, graphs, curves, etc. The results of classifying the forecast data by region, equipment type, time period, etc., are shown in Figure 3.

Figure 3 shows (1) a summary analysis by region. In order to facilitate the comparison between regions, the forecast should be classified and archived according to the location of the line, the rational use of the line between different regions should be analyzed, and the transmission pattern should be planned and adjusted. Whether the use of transmission lines is reasonable and whether the losses caused by abnormality are minimized are closely related to the cost of transmission lines. A more reasonable situation is to coordinate the transmission cost between each line and the cost of waste caused by an abnormality. In order to facilitate optimal planning and processing, abnormal prediction results and actual measurement results should be classified and archived according to regions to facilitate future use. (2) Summarize and analyze according to time. Line anomalies may vary with different time periods of the day, different days of each week and month, different months and seasons of each year, etc., which requires the forecast results to be aggregated by time. A high probability of anomalies usually accompanies high-power transmission. Transmission power is usually strongly correlated with time period, so it is necessary to summarize by time. Combined with location summary, dispatch to high-consumption areas when the line is idle, reducing the cost loss caused by line anomalies caused by high-power transmission. (3) Summarize and analyze according to the equipment type. Different types of equipment are related to the stable operation state, usage time, usage environment, and abnormal occurrence. Summarize according to equipment type to facilitate statistical analysis of abnormal conditions caused by a different equipment, and use different types of equipment under reasonable conditions.

Analysis of the cause of abnormal line status: the purpose is to provide advanced analysis, diagnosis, and processing services for abnormal data, so that relevant staff have time to do corresponding processing in advance and reduce the loss of manpower and physical costs caused by abnormal line status. In order to explain the abnormal situation, the abnormal situation needs to be explained, and the reason for the abnormal situation of the predicted line needs to be listed. The reason for this is to facilitate troubleshooting by staff. From the perspective of model prediction, not only the prediction accuracy of the model is required to be high, but also the selection of an algorithm with strong interpretability [12]. For some models, such as deep learning, the research results in data prediction are better, and they do not need to make explicit explanations and have better applicability.

### 2.2. Overview of ANN

Simplified neuron mathematical model: the artificial neuron network is an information processing system composed of the structure and function of the physiological real human brain neural network, as well as some theoretical abstraction, simplification, and simulation of some basic characteristics. It is an adaptive nonlinear dynamic system composed of many neurons through extremely rich and perfect connections.

The brain's neural network is a network composed of many highly interconnected neurons. It realizes the processing and storage of information through the interaction between neurons in the network. The brain neural network imitates the structure and function of the brain neural network and connects artificial neurons into a network according to certain structures and rules so that the connection weight of each neuron in the network changes according to certain rules to realize the learning or recognition of input patterns.

The brain neural network is a network composed of many highly interconnected neurons with statistical regularity. Due to the difficulty of physical implementation and the simplicity of calculation, the number of neurons that make up ANN is far less than that of a brain neural network. ANN is formed completely according to certain rules. Each neuron in ANN has the same structure, and in general, the actions of all neurons are synchronized in time and space.

**Figure 4:**
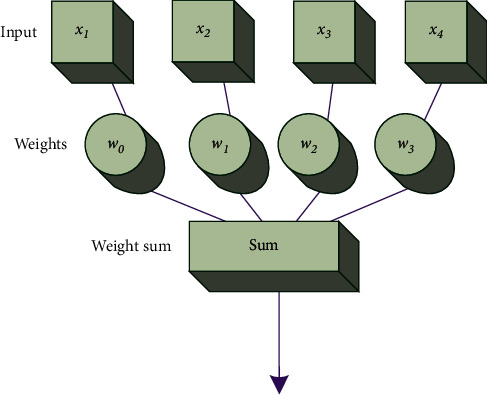
Mathematical model of artificial neuron.

The topological structure of ANN is generally divided into two types according to the flow direction of information: feedforward type and feedback type. ANN is also divided into a forward network and a feedback network. Some ANN has the same topology but has different functions and properties. This is because they have different study rules and work rules. The learning and working rules are the dynamic evolution rules of the connection weights between neurons in the network. In short, two main factors determine the properties of an ANN: one is the topology of the network; the other is the learning and working rules of the network. The formalized structural model of the artificial neuron is shown in Figure 4 [13].

In Figure 4, *x*_1_, *x*_2_, *x*_3_,…, *x*_*n*_ are input signals. Assumption: there are *n* neurons interconnected here, and the first neuron is used as the object. It inputs information to all other neurons *j*(*j*=1,2,…, *n*). The connection weight from the *j*th neuron to the *i*th neuron is denoted as *ω*_*ij*_.

Taking the quasi-linear unit model in the neuron model as an example, it uses continuous information as input and output. The biggest feature of this model is that the output function f(x) is in the form of

(1) $$\begin{array}{c} f\left(x\right)=\frac{1}{1+\text{exp}\left(-x+\theta_{i}\right)}. \end{array}$$

The total input *μ*_i_ of this model neuron *i* is given by

(2) $$\begin{array}{c} \mu_{i}=\sum_{j=1}^{n}\omega_{ij}x_{j}-\theta_{i}. \end{array}$$

In (2), *μ*_i_ is substituted into (3) as a variable *x*, and the output of neuron *i* can be calculated, as shown in

(3) $$\begin{array}{c} y_{i}=f_{i}\left(\mu_{i}\right). \end{array}$$

The output value y_i_ is also a continuous value. Quasi-linear unit models are widely used in BP networks [14].

### 2.3. BP Neural Network

**Figure 5:**
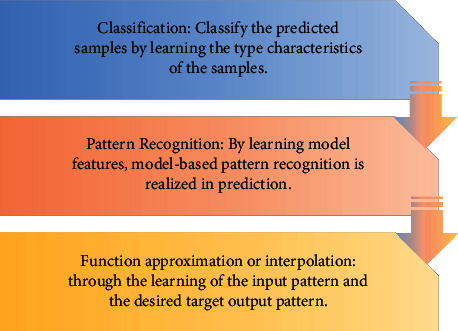
The main application scope of BP network.

Multi-layer feedforward network, also known as multi-layer feedforward network or multi-layer perceptron network, is one of the typical ANN models. It is also the earliest, most researched, and most widely used type of ANN model in pattern recognition and classification. The multi-layer forward network in which the back propagation algorithm is used for training is called a back propagation neural network, or BP network for short [15, 16]. In practical application, the BP network reflects the essential part of ANN at this stage. The main application scope of the BP network is shown in Figure 5.

**Figure 6:**
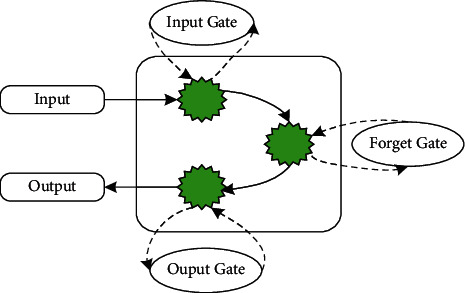
The training process of the BP algorithm.

The training process of the BP algorithm is shown in Figure 6.

Forward propagation of the working signal: the input signal is transmitted from the input layer to the output layer through the hidden unit, and a signal is generated at the output end. This is the forward propagation of the working signal. The weights of the network are fixed during the forward transmission of the signal. The state of neurons in each layer only affects the state of neurons in the next layer. If the desired output cannot be obtained at the output layer, the error signal is transferred to back propagation.

Error signal back propagation: the difference between the actual output and the expected output of the network is the error signal. The error signal starts from the output and propagates forward layer by layer. This is the back propagation of the error. During the back propagation of the error signal, the network weights are adjusted by the error feedback. The actual output of the network is closer to the expected output through the constant correction of the weights [17].

The back propagation process of the error and the forward propagation process of the signal together constitute the learning process of the BP algorithm. During the forward propagation of the signal, each neuron of the input layer is provided with input samples. The net inputs and outputs of the output layer and each hidden layer are computed. Further, the prediction result of the neural network is calculated. If there is a large error between the calculated prediction result and the expected output, the back propagation of the error is started. During the back propagation of the error, the input error is reversed in the output layer. The errors are back-propagated, and the weights are updated based on the errors at each layer. During this process, the weights will be continuously updated and adjusted. The learning process ends when the learning process reaches a preset number of cycles or the neural network output error is less than the specified threshold.

**Figure 7:**
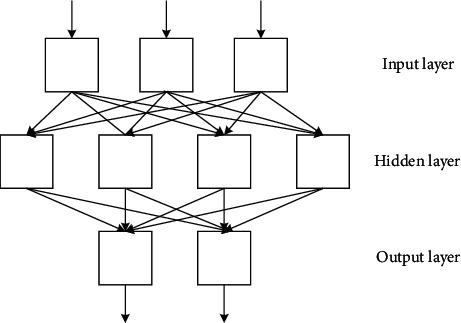
Schematic diagram of BP neural network.

A typical BP network is a three-layer feedforward hierarchical network, that is, an input layer, a hidden layer (also called an intermediate layer), and an output layer. A full connection is implemented between each layer, as shown in Figure 7.

In Figure 7, structurally speaking, the BP network is divided into an input layer, a hidden layer, and an output layer. Nodes at the same level are not related, and neurons at different levels propagate from front to back. Among them, the BP network corresponding to several nodes in the input layer can perceive several inputs. The output layer contains several nodes, and the BP network will have several output data. The number of nodes in the hidden layer needs to be adjusted or set according to the actual situation. The more the nodes in the hidden layer, the higher the accuracy of the result and the more time required [18]. Let the input layer be *M*. That is, there are *M* input signals, any of which is represented by *m*. The hidden layer is *J*. That is, there are *J* neurons, and any neuron is represented by *j*. The output layer is *P*. That is, there are *P* output neurons, and any neuron is represented by *p*. The synaptic weights of the input layer and the hidden layer are denoted by *W*_*mj*_. The synaptic weights of the hidden layer and the output layer are denoted by *W*_*jp*_. The input of the neuron is denoted by *u*, and the excitation output is denoted by *v*. The superscript of *u* and *v* represents the layer, and the subscript represents a neuron in the layer. It is assumed that the excitation function of all neurons uses the sigmoid function. The specific function formula between each layer is as follows:

The output function of the node in the hidden layer is shown in

(4) $$\begin{array}{c} b_{r}=f\left(\sum_{r=1}^{m}W_{ir}\cdot a_{i}+T_{r}\right)\left(r=1,2,\ldots ,u\right). \end{array}$$

The output function of the node in the output layer is shown in

(5) $$\begin{array}{c} c_{j}=f\left(\sum_{r=1}^{u}V_{ir}\cdot b_{r}+\theta_{j}\right)\left(j=1,2,\ldots ,n\right). \end{array}$$

Among them, the input layer node is *a*_*i*_. The connection weight between the input layer node and the hidden layer node *b*_*x*_ is *W*_*ir*_. The connection weight between the hidden layer node *b*_*x*_ and the output layer node *c*_*j*_ is *V*_*ir*_. *T*_*r*_ is the threshold of hidden layer nodes. *θ*_*j*_ is the threshold of the output layer node. *f*(·) is the transfer function, and usually the sigmoid transfer function is selected, as shown in

(6) $$\begin{array}{c} P={\left(1+e^{-x}\right)}^{-1}. \end{array}$$

The maximum value approaches 1, and the minimum value approaches 0. Typically, 0.5 is chosen as the threshold. Variables that are completely correlated are recorded as 1, and variables that are not correlated are recorded as 0. If the *P* value obtained by the operation is greater than 0.5, it indicates that the variable is highly correlated. If the obtained *P* value is less than 0.5, it indicates that the correlation between variables is low [19].

The specific BP network learning process is as follows:

Step 1 is to randomly assign a smaller value to *W*_*ir*_, *T*_*x*_, *V*_*ir*_, and *θ*_*j*_.
Step 2 is to perform the following operations for each pattern (*A*^(*k*)^,  *A*^(*k*)^) (*k*=1,2,…, *p*):
  (1) The value of *A*^(*k*)^ is input to the input layer node; that is, it becomes the input layer node activation value *a*_*i*_ and is calculated forward in turn.
  (2) The error calculation between the output *c*_*j*_ of the output layer node and the expected output value *c*_*j*_^(*k*)^ is shown in
    (7) $$\begin{array}{c} d_{j}=c_{j}\cdot \left(1-c_{j}\right)\cdot \left(c_{j}^{\left(k\right)}-c_{j}\right). \end{array}$$
  (3) The hidden layer nodes have assigned the error inversely, as shown in
    (8) $$\begin{array}{c} e_{x}=b_{x}\cdot \left(1-b_{x}\right)\cdot \left(\sum_{j=1}^{n}V_{rj}\cdot d_{j}\right). \end{array}$$
  (4) The connection weight W_ir_ between the input layer and the hidden layer nodes and the threshold T_r_ of the hidden layer nodes are adjusted:
    (9) $$\begin{array}{c} W_{ir}=W_{ir}+\beta \cdot a_{i}\cdot e_{r},\\ T_{r}=T_{r}+\beta \cdot e_{r}\left(0<\beta <1\right). \end{array}$$
  (5) Step 2 is repeated until the error E_AV_ becomes sufficiently small for j=1,2, ⋯, n, k=1,2, ⋯, p.
    (10) $$\begin{array}{c} E_{AV}=\frac{1/2\sum_{k=1}^{p}\sum_{j=1}^{n}{\left(c_{j}^{\left(k\right)}-c_{j}\right)}^{2}}{P}. \end{array}$$

Among them, *E*_*AV*_ is the training objective function. After repeated training, the error *E*_*AV*_ meets the accuracy required in the specific problem.

Two serious shortcomings of the BP network algorithm are the slow convergence speed and the existence of local minimum points in the objective function. These deficiencies affect the practical application of this network in many aspects. Therefore, many scholars have conducted extensive research on the learning algorithm of BP network and proposed some methods to improve the BP algorithm. The more common method is to add a momentum term. In the BP algorithm, the selection of the learning step *η* is very important. The larger the value of *η*, the faster the network converges, but too fast will cause oscillation. Although the small value of *η* can avoid oscillation, the convergence speed will be slow [20]. The easiest way to resolve this contradiction is to add a momentum term, as shown in

(11) $$\begin{array}{c} \Delta \omega_{mj}\left(n\right)=a\Delta \omega_{mj}\left(n-1\right)+\eta \delta_{j}\left(t\right)v_{m}\left(t\right). \end{array}$$

Adding the momentum term to the BP algorithm can not only fine-tune the correction of the weights, but also prevent the learning from falling into a local minimum. When deriving the BP algorithm, the learning parameter *η* is assumed to be constant. In fact, the *η* corresponding to different *ω*_*mj*_ is different.

## 3. Design of the Abnormal Displacement Detection Experiment of the Line

**Figure 8:**
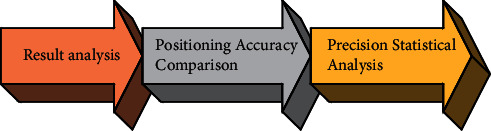
Comparison of the two algorithms.

The purpose is to implement a BDS based on BP neural network. It is used to monitor abnormal displacement problems in power lines. Geometric dilution of precision (GDOP) is used to measure the positioning error in the BeiDou positioning algorithm. The localized error is proportional to the size of the GDOP [21]. The BDS based on BP neural network and the conventional BDS are used to solve the satellite data. The results of the two solutions are compared. The main comparison items included are shown in Figure 8.

The results of the two positioning algorithms are analyzed by the difference between the positioning solution value and the actual value. The experiment performs static differential positioning on the data received by BDS based on BP neural network. In order to analyze the relative positioning accuracy of the algorithm more intuitively, the weighted least squares method is used for parameter estimation in the analysis process. Secondly, the GDOP of BDS is analyzed, taking the data collected by BeiDou in the Asia-Pacific region on April 1, 2019, as an example, a total of 160 epochs. The results are compared with the precise coordinates of the International GNSS Service (IGS) stations provided by the Scripps Orbit and Permanent Array Center (SOPAC) [22, 23].

The positioning accuracy of the two algorithms is compared. According to the algorithm flow of conventional BDS phase relative positioning and BDS phase differential positioning based on BP neural network, and using the data collected in the Asia-Pacific region, the epoch interval is set to 30 s, and the observation time is April 1, 2013., 28800 epochs. The positioning results of the two algorithm systems are obtained, and the positioning accuracy of the two positioning systems is analyzed and compared.

Statistical analysis of the accuracy of positioning results: root mean square (RMS) is the probability statistics of the deviation between the position of the monitoring point and its real position obtained by BDS modeling. Its value is not affected by the observation conditions. Analysis of the RMS can estimate the positioning accuracy of the positioning system [24]. RMS is defined as

(12) $$\begin{array}{c} \text{RMS}=\sqrt{\frac{V^{T}PV}{n-k}}. \end{array}$$

Among them, V^T^ represents the residual size of the observed value. *k* is the difference between the total number of observations and the number of unknowns. *P* represents the weight of the observation. When *P* is determined, the size of the error is related to the residuals of the observations. The unit weight error increases as the residual increases. The RMS can be used to analyze the goodness of the accuracy of the positioning results. The smaller the value, the better the quality of the observations and the higher the accuracy. The time period with better observation conditions at the Shanghai station is selected, and the simulation is performed 200 times with a sampling interval of 1 s. In both modes, the RMS of the positioning results of the monitoring station in the three directions of *X*, *Y*, and *Z* is recorded.

Neural network parameter design: the standard input of the neural network with the standard activation function is taken. The output data are limited to the range [0, 1]. The parameters (*X*, *Y*) in practical engineering applications have very large values and need to be converted into values in the [0, 1] interval. In addition, the region where the output is close to 0 or 1 is the saturation region of the network. Therefore, the output data range can be set to [0.2, 0.8] or [0.1, 0.9] to avoid the saturation region of the network.

Theoretically, GPS elevation fitting is a mapping from *Rn* (*n* = 2, representing the plane coordinates or geodetic coordinates of the GPS point) to *Rm* (*m* = 1, representing the abnormal or normal elevation of the GPS point). The classic BP neural network structure is a three-layer structure: input layer, hidden layer, and output layer. Therefore, the ideal BP neural network structure should be the structure of *A* × *M* × B. If the input layer takes two and the output layer takes 1, then the model 2 × *M* × 1 is the best choice. Among them, in the network construction test, *M* is first selected as 10. Because ten known points are selected in this engineering test, ten is selected, the 2 × 10 × l network is not redundant matching for known data, and ideal results can be obtained. For this purpose, a 2 × 10 × l network structure is chosen.

## 4. Experimental Results

Analysis of positioning results: in the experiment, static differential positioning is performed using the data collected by the BDS/GPS dual-mode system receiver developed by ComNav Technology Ltd. In order to analyze the positioning accuracy of GPS/BDS dual-mode carrier phase static relative positioning more intuitively, the weighted least squares method is used to estimate the parameters in the analysis process. BDS/GPS dual-mode systems require dual-mode receivers to have good observation conditions for satellites. When the number of satellites is less than 5, the dual-mode positioning solution cannot be performed. When various factors that affect the observation appear, the number of visible satellites becomes smaller, which in turn reduces the positioning accuracy. Assumption: the altitude angle of the satellite is 15°. In order to minimize the influence of error sources such as receiver clock error, ionospheric delay, and tropospheric delay, short baseline data are discussed, with a baseline length of about 1 km.

**Figure 9:**
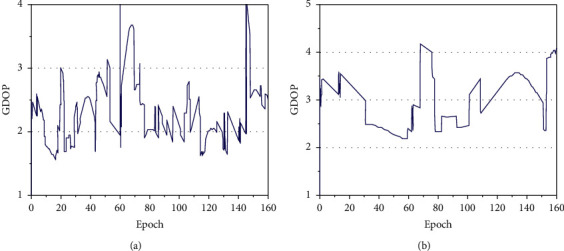
GDOP values of the two algorithms. (a) Conventional BDSGDOP value; (b) BDSGDOP value based on BP neural network.

Firstly, BDS and GPS satellites are analyzed. On April 1, 2018, from 12:00 to 13:20, the data collected by BDS and GPS in the Shanghai station in the Asia-Pacific region were taken as an example, with a total of 160 epochs. The number of visible satellites of BDS and GPS is similar. BDS is about 7–10, GPS is about 6–10, and GPS has more visible satellites than BDS. This is because the current BDS satellite launch volume has not reached the full planned number of GPS satellites. The GDOP values of the two algorithms are shown in Figure 9.

Figures 9(a) and 9(b) show the simulated GDOP values with a different BDS positioning. The GDOP values obtained by conventional BDS are small, all in the range of less than 4. The geometric space distribution of positioning satellites is improved after the introduction of the BP neural network. The reduction of the GDOP value enhances the reliability and availability of satellite positioning and meets the accuracy requirements. The BDSGDOP value based on the BP neural network is smaller than that of the conventional system, and the positioning accuracy is better.

**Figure 10:**
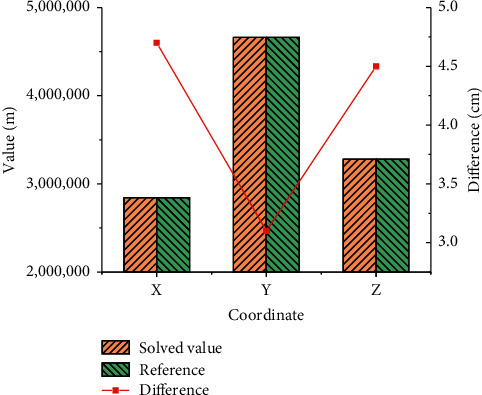
Comparison of positioning results and reference values.

The solved data results are compared with the exact coordinates of the IGS station. The localization result of BDS short baseline vector based on BP neural network is shown in Figure 10.

In Figure 10, *X* represents the horizontal displacement data on the plane in the three-dimensional space, *Y* represents the vertical displacement data on the plane, and *Z* represents the vertical displacement data. The unit of solution value and reference value is *m*, and the unit of difference value is cm. The BDS displacement monitoring method based on BP neural network is basically consistent with the coordinate values obtained by using the IGS integrated track. The effect of the positioning results is more ideal with the continuous improvement of BDS and the further improvement of the precision of the corresponding precision track products.

**Figure 11:**
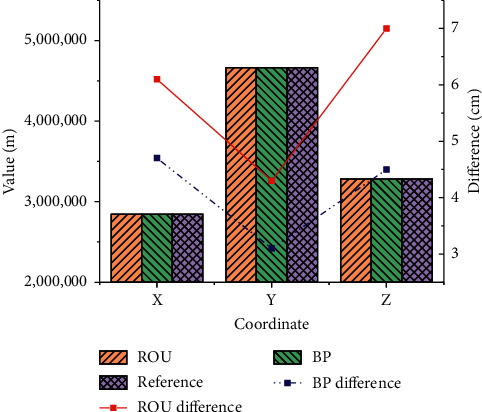
Comparison of the positioning results and reference values of the two algorithms.

Comparison of conventional BDS and improved BDS positioning accuracy: according to the algorithm flow of single GPS carrier phase relative positioning and BDS/GPS dual-mode carrier phase differential positioning, the positioning programs are written, respectively. Likewise, the data collected at the Shanghai station in the Asia-Pacific region are used. The epoch interval is set to 30 s. The observation time is April 1, 2018, with 2880 epochs throughout the day. In the navigation and positioning results, the accuracy statistics obtained by BDS based on BP neural network and conventional BDS are compared, as shown in Figure 11.

In Figure 11, ROU represents conventional BDS. The accuracy of the measured data positioning results of the two systems has reached the cm level. The difference is small between the processing results of the two modes. Among them, the *Z* direction accuracy has the largest difference, which is 2.5 cm. The introduction of the BP neural network has improved the spatial combination structure, and the positioning results in the three directions of *X*, *Y*, and *Z* are all better.

**Figure 12:**
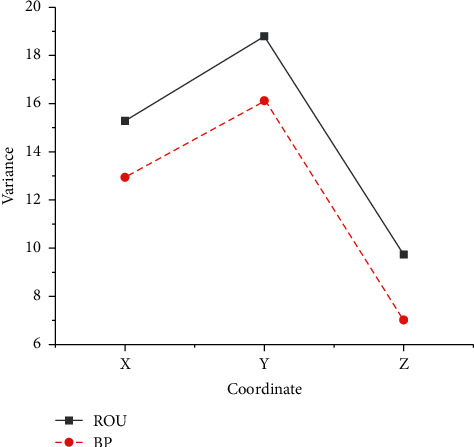
Analysis of error variance.

In order to better compare and analyze the positioning accuracy of the two systems, the error variance is obtained by the mean of the deviation of the positioning results, which is used as one of the indicators to reflect the accuracy of the positioning results. The numerical statistics results are shown in Figure 12.

In Figure 12, compared with the conventional BDS, the variance value of the BDS based on the BP neural network is smaller, indicating that the phenomenon of poor satellite geometric distribution is less, the static differential positioning result obtained by the least squares method has less fluctuation, jump is small, and the results converge well. The positioning accuracy of the system is improved compared with the conventional system.

**Figure 13:**
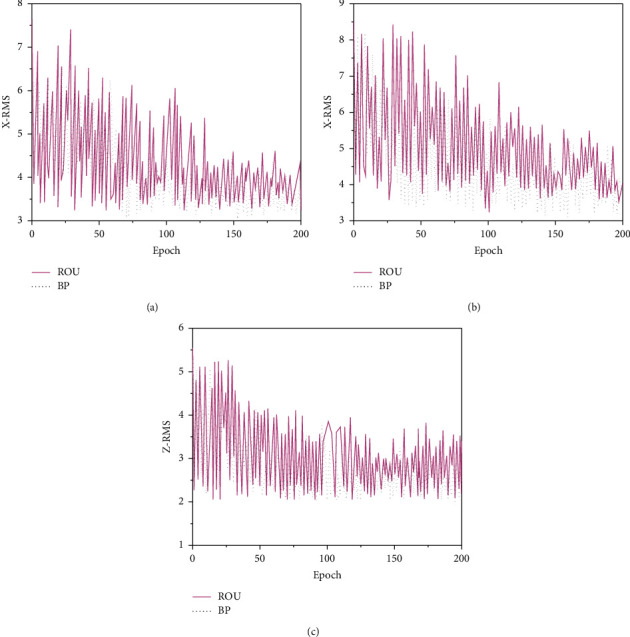
Positioning RMS of the two systems in different directions. (a) RMS in *X* direction; (b) RMS in *Y* direction; (c) RMS in *Z* direction.

The RMS of the positioning results of the monitoring station in the *X*, *Y*, and *Z* directions in the two modes is shown in Figure 13.

Figures 13(a)–13(c) are the displacement errors of the coordinates of the monitoring station in the *X*, *Y*, and *Z* directions when the two systems are used for positioning, respectively. When the simulation parameters are the same and the operation is normal, different satellite systems are tracked, and the RMS of the positioning result obtained by observing the conventional BDS fluctuates greatly. The RMS value of BDS displacement based on the BP neural network is smaller, and the change is gentle. As the number of epochs increases, the number of simulations increases, and its value becomes more convergent. Additionally, the RMS value obtained by the system operation is smaller than that of the conventional system, indicating that the BDS observation quality based on BP neural network is better.

**Figure 14:**
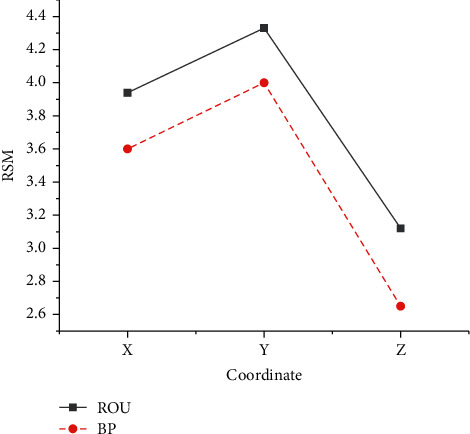
Statistics and comparison of RMS values.

The RMS means of the positioning results of the two systems is shown in Figure 14.

In Figure 14, the RMS average of the BDS localization results based on the BP neural network is smaller than that of the conventional BDS. Among them, the RMS of the positioning result error of the former is not more than 4 cm, which reflects that the former has effectively improved the positioning data processing method of the latter. The positioning results obtained by this system are more stable and more reliable. From the point of view of mathematical statistics, the error fluctuation range of BDS positioning results based on BP neural network is relatively small, and it is better in positioning accuracy.

Bao et al. [25] used wavelet analysis theory and proposed a wavelet threshold filtering method based on empirical mode decomposition to detect overhead high-voltage transmission line faults. For overhead high-voltage transmission line faults, it should be noted that there are three types of noise: Gaussian white noise interference, periodic narrowband interference, and impulse interference. The appearance of these noise signals will increase the leakage current error of high-voltage transmission lines, thus interfering with fault detection. Therefore, the interference noise on-site must be eliminated or suppressed first, and the positioning result obtained by the system is more stable and more reliable. From the point of view of mathematical statistics, the error fluctuation range of BDS positioning results based on BP neural network is relatively small, and it is better in positioning accuracy.

## 5. Conclusion

Power lines are the most important link in the transmission of electrical energy from power grids to users. In order to reduce the failure of power lines and ensure the safety of people's lives and property, in addition to strengthening protection measures from personnel management, technically, it also puts forward higher requirements for abnormal displacement monitoring of power lines. It is urgent to develop a reliable and stable abnormal displacement monitoring and positioning system. The satellite positioning system performs positioning, timing, and monitoring of users on the Earth through the time, position, speed, and other signals broadcast by satellites.

BP neural network is used to optimize the BDS. The improved BDS and the unimproved conventional BDS are used to solve the collected correlation data. The accuracy of the solutions of the two systems is compared by designing experiments. The experimental results show that the GDOP values obtained by conventional BDS are small, all within the range of less than 4. After the introduction of the BP neural network, the geometric space distribution of the positioning satellites is improved, the GDOP is reduced, the reliability and availability of the satellite positioning are enhanced, and the accuracy requirements are met. The accuracy of the measured data positioning results of the two systems has reached the cm level. There is not much difference between the processing results of the two modes. Among them, the *Z* direction accuracy has the largest difference, which is 2.5 cm. The introduction of BP neural network has improved the spatial combination structure, and the positioning results in the *X*, *Y*, and *Z* directions are all better. The RMS average of BDS localization results based on the BP neural network is smaller than that of conventional BDS, and the RMS of the former's localization result error does not exceed 4 cm. This reflects that the former has effectively improved the positioning data processing method of the latter. From the perspective of RMS, the simulation results obtained by conventional BDS have large fluctuations in RMS. The RMS value of BDS displacement based on the BP neural network is smaller, and the change is gentle. With the increase in simulation times, its value is more convergent, indicating that the quality of BDS observation based on the BP neural network is better. Due to limited energy, the study did not consider the monitoring accuracy of the system under an abnormal climate. In the future, this aspect will be further explored.

## Data Availability

The data used to support the findings of this study are available from the corresponding author upon request.
